# Supplementary material for: Diagnostic performance of basal cortisol level at 0900-1300h in adrenal insufficiency
Source: PLoS One. 2019 Nov 18;14(11):e0225255. doi: 10.1371/journal.pone.0225255 (PMC6860436; doi:10.1371/journal.pone.0225255)
Supplement: Attachment — Submitted filename: responses to reviewers.docx [file pone.0225255.s001.docx]

**Responses to reviewers**

We thank the reviewers for their careful reading of the manuscript and their constructive comments. We have taken all the comments to improve and clarify the manuscript. Please find below a detailed point-by-point response to all comments (reviewers’ comments in bold black, our replies in non-bold black).

Since the reordering and restructuring of the manuscript was substantial, we have written bullet points of our major changes to the manuscript. Also, line numbering was referred to the revised manuscript without track changes.

- The manuscript has been edited by English language editing service (please see the attached certificate from AJE)
- In order to reduce the over estimation of AI prevalence, the cut-off levels to diagnose AI for serum cortisol post-ACTH stimulation test was modified to 420 nmol/L (instead of 500 nmol/L) according to proposed cut-point for second generation Cortisol II assay
- All the units have been changed to SI with conventional units in parentheses
- Accordingly, 29 patients were excluded from the cohort, as the inclusion criteria was changed to patients with morning cortisol between 86-420 nmol/L
- Statistical power for our final sample size was still more than 80%
- The main concept and results are still the same
- New Table 2 was created to demonstrated uni- and multivariate analyses of basal and morning cortisol
- New Table 3 for each cut-off levels was created, and multiple cut-off levels were added with the interval of 25 nmol/L or lower
- New Figure 1 was created to demonstrate and compare multiple serum cortisol values between patients with and without AI

**Reviewer#1**

Reviewer #1: Manosroi et al. have examined the diagnostic performance of the time 0 cortisol drawn during a standard or low dose ACTH stimulation test undertaken between 0900h-1300h compared to a 0800h cortisol, to diagnose adrenal insufficiency in a patient cohort from Thailand. They conclude that a cortisol drawn between 0900h-1300h has a higher diagnostic performance than an 0800h cortisol in diagnosing adrenal insufficiency. Specific comments:

**1. The main limitation of the study is the inappropriate use of a cortisol cut-off of 500 nmol/L in the ACTH stimulation test with the Roche Electrosys Cortisol II assay. Two separate publications have suggested a much lower cut off of 350 nmol/L (12.7 mcg/dL, Kline et al. Clin Biochem 2017) and 374 nmol/L (13.6 mcg/dL, Raverot et al. Ann Endocrinol 2016). Therefore using the 500 nmol/L or 18 mcg/dL as generically recommended in Ref 3 will markedly over-estimate the true prevalence of adrenal insufficiency in this cohort. It is now well established that assay-specific reference ranges are required to be used (El-Farhan et al. Clin Endocrinol 2013). This then makes the interpretation of any relationship between diagnosed adrenal insufficiency and basal cortisol (be it at 0800h or 0900-1300h) almost meaningless.**

Thank you for this very helpful comment.

After thoroughly reviewed the literatures regarding the assay-specific cut-off levels for cortisol II assay, we totally agree with your comment (Javorsky et al. [*J Endocr Soc*](https://www.ncbi.nlm.nih.gov/pmc/articles/PMC6552067/). 2019 Apr 15; Klein et al. *Clin Biochem* 2017; El-Farhan et al. [*Clin Endocrinol (Oxf).*](https://www.ncbi.nlm.nih.gov/pubmed/22994849)2013 May; Vogeser et al*. Clin Chem Lab Med*. 2017). Endocrine Society Guideline for adrenal insufficiency also stated that the appropriated cut-off for ACTH stimulation tests should be assay-specific. After discussing with all the authors, we agreed to adjust the diagnostic criteria of adrenal insufficiency by using a lower peak serum cortisol of 420 nmol/L (15.1 µg/dL) instead of 500 nmol/L which based on the data from El-Farhan et al. [*Clin Endocrinol (Oxf).*](https://www.ncbi.nlm.nih.gov/pubmed/22994849)2013. This cut-off was derived from the lower reference limit at 30 min after ACTH stimulation standardized by GC-MS method. Therefore, this could avoid the overdiagnosis prevalence of adrenal insufficiency in our population and our main results could be applied in real practice

Therefore, the analyzed data have been changed accordingly including the number of total populations. We have changed our inclusion criteria to those who had 0800hr serum cortisol between 83-420 nmol/L (3-15.1 µg/dL) instead of 83-500 nmol/L (3-18 µg/dL). Therefore, only 29 patients were excluded from the cohort. The prevalence of adrenal insufficiency was decreased from 23.8% to 21.5% and there were some changes in the proposed cut-off levels as well. However, the main idea and results are still the same. Also, the new acquired co-variate adjusted ROC for serum basal cortisol was higher than the previous result. Also, the calculated power for the new sample size was still adequate (>80%) (Bujang et al. [J Clin Diagn Res](https://www.ncbi.nlm.nih.gov/pmc/articles/PMC5121784/). 2016 Oct). We hope that you will agree with our new peak cortisol cut-off levels for ACTH stimulation tests.

Using 500 nmol/L for peak cortisol level is the number we normally use in our institution according to the Endocrine Society Guideline. We hope that we will change our practice of using the new cut-off levels for peak cortisol after ACTH stimulation soon. Thank you.

**2. None of the cortisol data are depicted, the only figure is the ROC curve. Could the authors clarify if the criteria for a passed test was a cortisol of >18 mcg/dL at either 30 or 60 minutes, with the same criteria for both low dose and high dose tests?**

Thank you for raising this point. We have provided the data of all cortisol levels categorized by adrenal insufficiency or no adrenal insufficiency status in box-plot diagram in **Fig 1(new).**

The Endocrine Society Guideline of adrenal insufficiency recommended the same cut-off for peak cortisol after ACTH stimulation tests of both low and high dose tests. Therefore, the authors came to the conclusion that we will use the same criteria for adrenal insufficiency for both low and high dose ACTH test are the same which is 420 nmol/L. We have added that in the manuscript in **L99-100.** Also, we have used ACTH dose (low or high dose) as one of the covariates and were adjusted in multivariate model in order to reduce the variability of the dose ACTH used.

**3. Generally when establishing reference intervals, the 95% confidence interval in a normal population is used, so the upper cut point would be a 97.5% CI. Why did the authors use a >99% sensitivity/specificity when proposing their upper and lower cut points?**

Normally, the 95%CI was used to estimate the range of the point estimate or to document the percentile. The way we chose each cut-off level is not depend on the 95%CI of overall patients in the cohort. We manually chose a very high sensitivity (>99%) for upper cut-off level to rule-out adrenal insufficiency as this is a lethal condition if we misclassify them. Likewise, the lower cut-off to rule in adrenal insufficiency should have a very high specificity, because to prescribe physiologic dose of corticosteroid may do harm to the patients if we over-diagnose them. Therefore, we finely adjusted each cut-off value level by level with the interval of 25 nmol/L or lower, as appropriate. Therefore, each cut-off level has its own sensitivity, specificity and 95%CI.

We have revised **Table3 (new)** and provided the data of 95%CI for each proposed cut-off sensitivity and specificity. We also have discussed the reason why we chose a very high sensitivity and specificity for each cut-off levels in **L220-230 (discussion section).**

**4. The findings are of interest, and the potential flexibility of being able to usefully interpret a cortisol drawn any time before 1300h would be beneficial for patients and clinicians alike. The main difference in the study between the "morning" cortisol and "basal" cortisol was the upper cut off of 16 vs 12.5 mcg/dL. My suspicion is that many of the patients who failed the ACTH stimulation test whose "morning" cortisol was between 12.5 mcg/L and 16 mcg/L probably in fact had normal function if an appropriate stimulated cortisol cut-off was applied.**

Thank you for raising this issue.

We have applied the new cut-off level for stimulated cortisol and the new proposed upper cut-off to rule-out AI in our study were >350 nmol/L (12.6 µg/dL) for 0800h morning cortisol and >337 nmol/L (12.2 µg/dL) for serum basal cortisol. Comparing to our previous results, the upper cut-off for 0800h morning cortisol has decreased from 440 to 350 nmol/L.

Further analysis

|  | **Serum morning cortisol** | |
| --- | --- | --- |
| **Basal cortisol** | >350 nmol/L | ≤350 nmol/L |
| >337 nmol/L | 14 | 91 |
| ≤337 nmol/L | 31 | 306 |

The new upper cut-off for both morning and basal cortisol (350 and 337 nmol/L) showed smaller interval compared to our previous one (440 and 347 nmol/L)

There were 91 patients who had levels between 337-350 nmol/L. Based on our analysis, only one (1/91) patient in this group was truly diagnosed with adrenal insufficiency and the other 90 patients had normal adrenal function. This could presume that our proposed cut-off level to rule out AI particularly from basal cortisol had fairly high accuracy.

**Reviewer#2**

**Manosroi et al. present an interesting piece of research. In my opinion, it can add substantial guidance to the community, but it needs thorough revision first.**

Thank you for the positive and encouraging comments.

**Minor:**

**1) L 50: Please provide cortisol reference ranges in international units at least the first time they appear in the text.**

Thank you. We have changed all units in the text to SI with conventional units in the parentheses.

**2) L56: remove “there is” as it is unnecessary**

It was removed as suggested.

**3) L73 and 74: Rewrite to ….estrogen, patients with suspected congenital adrenal hyperplasia…**

Thank you. The sentence has been rewritten as suggested.

**4) L99: Rewrite “…history of personal using of…” to “…history of personal use of…”**

The sentence has been rewritten as suggested.

**L105 & 118 : Make sure to use the correct temporal form, multiple uses of past tense although present would be appropriate, for instance “..characteristics were depicted in Table 1.” Instead of “characteristics are depicted”.**

We have corrected the sentence as suggested. Thank you.

**5) Discussion: Although not a native speaker myself, I detected various semantic, and grammar mistakes in this section. They fall in the same category as those above. Please, revise your manuscript carefully.**

Thank you. According to this issue, we apologize for all the grammatical mistakes and the revised manuscript has been edited by the English language editing service (please see the attached certificate from AJE).

**Major:**

**Table 1: Numbers of categorical variables do not add up to 471. For instance, underlying diseases adds up to 268 only. Please provide on missing data. It is ok if this piece of information is retrospectively not retrievable anymore, as long it is clearly stated.**

Thank you for noticing this.

Reviewer#1 has raised issue regarding the diagnostic cut-off point for Cortisol II electrosys assay We have thoroughly reviewed multiple literatures about this second generation assay and the specific cut-off levels for ACTH stimulation test which are lower than the usual cut-off points (Javorsky et al. [*J Endocr Soc*](https://www.ncbi.nlm.nih.gov/pmc/articles/PMC6552067/). 2019 Apr 15; Klein et al. *Clin Biochem* 2017; El-Farhan et al. [*Clin Endocrinol (Oxf).*](https://www.ncbi.nlm.nih.gov/pubmed/22994849)2013 May). If the previous cut-off for cortisol (500 nmol/L) was used, this will markedly overestimate the real prevalence of adrenal insufficiency. Therefore, we agreed with Reviewer#1 and we have modified the diagnostic criteria for adrenal insufficiency by using the peak cortisol cut-off at 30 or 60 min at 420 nmol/L (15.2 µg/dL) instead of 500 nmol/L (El-Farhan et al. [*Clin Endocrinol (Oxf).*](https://www.ncbi.nlm.nih.gov/pubmed/22994849)2013 May).

Therefore, the data have been modified accordingly and the new number of all population were 442 patients as only 29 patients were excluded. Some numbers have been changed but the main idea and results are still the same. Also, the power of our new sample size is still >80% (Bujang et al. [J Clin Diagn Res](https://www.ncbi.nlm.nih.gov/pmc/articles/PMC5121784/). 2016 Oct).

We also added the data in **Table 1** for patients with no known underlying disease and other diseases. Please note that the add up numbers are higher than number of total populations as some patients may have more than one underlying disease. Also, other demographic data have been revised. There was no missing value for demographic variables. For biochemical variables apart from serum cortisol (e.g. albumin, cholesterol, creatinine), the missing variables of more than 5% were dealt with multiple imputation analysis as stated in **L120-121**.

**L193 Rewrite “Of note, the different could be” to “Of note, the difference could be”**

Thank you. The sentence has been rewritten.

**Table 2: Authors should provide cortisol in international units in the legend to facilitate understanding for readers from Europe. Also, it would be prudent extend the table to include various ranges of cut-offs (maybe in steps of 2ug/dl?). This way readers can judge for themselves which cut-off would be optimal for their need.**

As per Plos One manuscript guideline, we have changed all units to SI units with conventional units in parentheses.

We have modified an old Table 2 to the (new) **Table 3** which included multiple cut-off values with the interval of 25 nmol/L (~1 µg/dL) or lower. 95%CI of sensitivity, specificity, adjusted AUC and non-adjusted AUC for each cut-off level were provided.

**What I liked is that numbers of true negatives, true positives etc. were presented. This really helps to find out how many patients could potentially be misclassified, if the proposed cut-off were applied in practice.**

Your encouraging comment is greatly appreciated. Thank you.

**Authors should include a discussion how they arrived at the proposed cut-offs. Were they stated *a priori* or *a posteriori*?**

We have added the statement in the statistical analysis section **L116-118**. Also, in the discussion section, we have stated the reason why we have to choose the upper cut-off with the highest sensitivity and the lower cut-off with the highest specificity in **L220-230.**

**Furthermore, it is misleading to state an AUC of 0.82 in the abstract that is derived from figure 1, but nowhere presented (such as in table 2). Additionally, discussion should include a statement on the predictive abilities of ROC curves themselves. In case of rare events (what an AI in clinical practice gladly is), it tends to give overoptimistic results. Also, the overall AUC might not be what a reader is interested in, as it can be deceptive. Although AI is rare but potentially deadly, a clinician wants to minimize the numbers of false negatives. Therefore, it is important that these figures are presented in table 2.**

We have created (new) **Table 2** with data of both univariate and multivariate analyses of diagnostic accuracy for serum morning and basal cortisol. Also, both covariate-adjusted and non-adjusted AUCs were demonstrated in **Table 2**.

The discussion regarding the diagnostic accuracy based on ROC alone was added on **L207-211** and **L223-227**.

**I am honestly unsure how creatinine and serum cholesterol could affect the level of cortisol. Authors should either remove these variables form analysis or clearly state why they included them. I might rather be sensible to include the dose of ACTH (LDT vs. HDT) used as a covariate.**

Thank you for raising this issue.

We agree with you on your helpful comment on the ACTH dosage as a covariate. As the ACTH dosage itself could affect serum cortisol level, therefore, we have re-analyzed the data and adjusted for ACTH dosage in multivariate analysis as suggested.

The associations between serum cholesterol and adrenal insufficiency in cirrhotic patients have been reported in multiple literatures including human and animal studies with varying results. However, there was no report of this issue in non-cirrhotic patients (Park et al*. Medicine.* 2018;97(26); Spadaro et al. *Scandinavian journal of gastroenterology*. 2015;50(3); Ouweeneel et al. *Atherosclerosis*. 2017;261). As cholesterol is the precursor for glucocorticoids synthesis ([Bochem](https://www.ncbi.nlm.nih.gov/pubmed/?term=Bochem%20AE%5BAuthor%5D&cauthor=true&cauthor_uid=23511897)et al. [*J Lipid Res*](https://www.ncbi.nlm.nih.gov/pmc/articles/PMC3646470/)*.* 2013 Jun), we assume that cholesterol levels may cause an interference with cortisol levels. We have stated this issue in the discussion part (**L256-258**).

However, multiple studies had reported normal findings of adrenal function in chronic renal failure patients, physiologic changes of serum cortisol have been reported in multiple studies (Clodi et al. *Am J Kidney Dis.* 1998;32(1); Raff et al. *Endocr Connect.* 2013;2(1)). Serum half-life of cortisol is prolonged in chronic renal failure and both elevated and normal cortisol levels had been reported (Bacon et al. *The Johns Hopkins medical journal*. 1973; Nolan et al. JCEM. 1981;52(6); Sianmopoulous et al. *Peritoneal dialysis international.* 1990;10(2)). Therefore, in those with poor eGFR (high serum creatinine), serum cortisol may be altered. So, we have used serum creatinine as one of the covariates. We also have stated this issue in the discussion part (**L256-258**).

**Furthermore, results from unadjusted analysis are not presented. Please include them in a separate column in table 2.**

The new **Table 3** for the unadjusted and adjusted data (AUCs) was created as suggested. Thank you.

**L202 I assume that Cortisol II stands for second generation cortisol immunoassays. Please elaborate more on the differences between assays.**

Thank you. We also discussed more about the differences between assays and the application of the proposed cut-off levels in other assays in the discussion section. (**L242-250**)

**L 227 and 228 “Another limitation was the exact time at which the basal cortisol and ACTH stimulation had been tested were not provided but most of the patients were tested before noon.” This sentence questions the whole validity of the study. Please present the testing times in table 1. Table 1 should also include two additional columns splitting the population into 2 groups according to a morning cortisol group and a basal cortisol group.**

Thank you for raising this issue.

We apologized that the data of the specific time point at which basal cortisol was tested were not available in our cohort. The time was recorded in the range between 0900h and 1300h. Therefore, we put this issue as one of our limitation.

However, one study in year 2019, also demonstrated a high sensitivity (>95%) of serum cortisol testing in the time range between 0800h-1200h (Mackenzie et al. *Clin Endocrinol*. 2019). The discussion regarding this limitation was added in **L277-281**. Therefore, we presumed that to test cortisol with our proposed time range still has validity. We plan to perform a prospective study regarding multiple time testing if serum cortisol in the future.

Each patient in our cohort had been performed both basal and morning cortisol levels as stated in the ACTH stimulation testing protocol section. Therefore, we cannot categorize the patients into 2 groups as you suggested. To performed both tests in the same patients support our results validity as this could reduce the confounders which occur from different patients.

**If I am correct, there was no reason stated why the data should be available only upon request.**

After discussing with other authors, the was no restriction to our data access. We have edited that in the submission form. The URL for the data access is <https://drive.google.com/open?id=1XutNq1CD57xqjOInx_VM46LLXCCkEaDa>

Thank you.
