# Supplementary material for: Diagnostic performance of basal cortisol level at 0900-1300h in adrenal insufficiency
Source: PLoS One. 2019 Nov 18;14(11):e0225255. doi: 10.1371/journal.pone.0225255 (PMC6860436; doi:10.1371/journal.pone.0225255)
Supplement: Attachment — Submitted filename: responses to reviewer.docx [file pone.0225255.s002.docx]

**Response to reviewers**

**Reviewer #1**: Manosroi et al. have undertaken a substantial revision of the manuscript and it is significantly improved. Some issues remain to be addressed:

**1. Ref 11 (El-Farhan et al.) did not use the Roche cortisol II assay, they used the older Roche (cortisol I) E170 assay. Their cut-off of 420 nmol/L is based on GCMS. The papers cited by the authors which did use cortisol II, namely Javorsky et al. and Kline et al. reported cortisol cut offs of 400 nmol/L and 350 nmol/L respectively (and a third study previously mentioned by this reviewer, Raverot et al. was 374 nmol/L). Therefore it is puzzling why the authors chose 420 nmol/L as their cortisol cut-off. Furthermore, since the data are from 2010-2017, it seems unlikely that the authors' laboratory used a cortisol II assay for this entire period.**

**Response:**

We would like to apologize for our mistake in stating the wrong generation of assay used for cortisol testings in the manuscript. After we have double checked with the former head of the Endocrinology Laboratory at our institution, the Elecsys Cortisol II assay has been used since January 2016 until present time. Therefore, the period between January 2010-December 2015, the cortisol tests were performed by electrochemiluminescence immunoassay using an Elecsys model 1010 (Roche Diagnostic, Laval, Quebec) which is an older assay. Please find the **attached certify letter** from the former head of Endocrinology laboratory from our institution.

As the serum cortisol in the main population was performed by first-generation assay, we decided to exclude those with second-generation assay. The overall population were 471 patients. There were 55 patients excluded and 416 patients included. Also, as the assay used was the first generation, the conventional cut-off (peak cortisol <500 nmol/L) for peak cortisol after ACTH stimulation test was used to diagnose AI and we have included those with indeterminate serum cortisol between 83-499 nmol/L (Bornstein. [*J Clin Endocrinol Metab.*](https://www.ncbi.nlm.nih.gov/pubmed/26760044) 2016 Feb). Although, the total sample size has reduced, the statistical power is still more than 80% (Bujang*.* [*J Clin Diagn Res*](https://www.ncbi.nlm.nih.gov/pmc/articles/PMC5121784/). 2016 Oct)

We have re-analyzed all the data according to the new cut-off level to diagnose AI (peak cortisol <500 nmol/L).

- All the Tables (**Table 1-3)** and Figures **(Figure1 and 2)** have been modified accordingly.
- In the method section, the period of data collection **L72** and **75** has been changed. The method of cortisol assay used in the study has been modified (**L85-86**)
- In the ACTH stimulation testing protocol section, the indeterminate cortisol levels have been adjusted to level 83-499 nmol/L (**L91**)
- In the definitions section, the peak serum cortisol for AI diagnosis has been changed to >500 nmol/L (**L103-105**)
- In the discussion section, **L248-256** have been modified.
- Minimal changes of some values in baseline characterisitics, results and discussion section.
- Only minimal changes in upper and lower cut-off levels were observed with the same sensitivity and specificity.
- The main concept of the manuscript is still the same.

We again apologized for our mistake and misunderstanding regarding the assay used in the manuscript which bring to some confusions. We hope that to include only those with older assay would benefit in terms of data homogeneity. Thank you.

**2. I think it is misleading if not frankly incorrect to state that their proposed cortisol cut-offs demonstrated that the basal cortisol drawn later in the day had a higher diagnostic performance than a 0800h cortisol. Firstly neither the lower nor upper proposed cut-offs are significantly different from each other, when taking into account the cortisol assay CVs. The lower cut off of 89 vs 86 nmol/L and the upper cut-off of 350 vs 337 nmol/L are well within even a 5% CV boundary.
Secondly, even if one takes these as different numbers, on the analysis performed there was 1 false positive and 1 false negative in each group, with identical sensitivity at the upper cut-off and specificity at the lower cut-off.**

**Response:**

The reason why we stated that basal cortisol had higher diagnostic performance than morning cortisol was based on the adjusted area under ROC (**Fig 2**). Area under ROC is the degree of agreement between the index test (basal or morning cortisol) and the reference standard (ACTH stimulation test) in which basal cortisol level showed higher rate of agreement to ACTH stimulation tests than morning cortisol (Florkowski. [*Clin Biochem Rev*](https://www.ncbi.nlm.nih.gov/pmc/articles/PMC2556590/)*.* 2008 Aug). This AuROC in **Fig2** was calculated by the overall values for basal and morning cortisol, not for just the specific cut-off level.

The upper and lower cut-off levels we have proposed for basal and morning cortisol per se did not provide data on diagnostic accuracy for the whole values. The proposed cut-off level that we have chosen to report is just the spot on the ROC curve which gave the highest sensitivity or specificity for basal or morning cortisol. Therefore, these spots cannot demonstrate the whole picture of diagnostic accuracy unlike AuROC in **Fig2**. (Unal. [*Comput Math Methods Med*](https://www.ncbi.nlm.nih.gov/pmc/articles/PMC5470053/)*.* 2017)

We agree with you that our proposed cut-off levels between basal and morning cortisol were almost the same number which gave the same sensitivity and specificity. However, after statistical analysis by comparing an adjusted AuROC between the lower cut-off for morning (<90 nmol/L) and basal cortisol (<85 nmol/L) as shown in the table and figure below, basal cortsol showed statistically higher AuROC (diagnostic accuracy) than morning cortisol (p<0.01). These data were not shown in the manuscript.

|  | **AuROC** | **p-value** |
| --- | --- | --- |
| Morning cortisol | 0.65 | 0.01 |
| Basal cortisol | 0.71 |  |

Figure demonstrated AuROC between proposed lower cut-off level for basal and morning cortisol

Likewise, the upper cut-off levels for basal (>350 nmol/L) and morning cortisol (>380 nmol/L) demonstrated significantly different in diagnostic accuracy as shown in the table below.

|  | **AuROC** | **p-value** |
| --- | --- | --- |
| Morning cortisol | 0.68 | 0.003 |
| Basal cortisol | 0.76 |  |

Figure demonstrated AuROC between proposed upper cut-off level for basal and morning cortisol

Also, when looking at the absolute number of patients using these cut-off levels for morning and basal cortisol, nearly 10% and 30% of the patients (true positive and true negative numbers) could bypass the ACTH stimulation tests (as mentioned in the discussion section **L241**).

Based on the abovementioned data, these may be inferred that serum basal cortisol and the proposed cut-off levels have higher diagnostic performance than serum morning cortisol.

**Point 1 is essential to resolve in my opinion - the cut-off of 420 nmol/L is still at least 20 nmol/L higher than any published data on the cortisol II assay in the ACTH stimulation test, if this was in fact the assay used throughout the study period.**

We have addressed and clarified this issue on **question#1**.

**Taken on face value with the current data set, the conclusion of the study should be that a basal cortisol taken any time during the morning up to 1200h (it is unclear how many were done between 1200h and 1300h) provides useful information in assessing the HPA axis integrity, though the cut-offs are not significantly different from the 0800h cortisol.**

Thank you for raising this issue, we have discussed this issue in the first paragraph of **question#2**.

**Reviewer #2: My concerns have been addressed. I appreciate the extensive work of the authors in regard to language, statistics and readability.**

Thank you. We are really appreciate all your helpful comments which help improved our manuscript.
