# Supplementary material for: Diagnostic performance of basal cortisol level at 0900-1300h in adrenal insufficiency
Source: PLoS One. 2019 Nov 18;14(11):e0225255. doi: 10.1371/journal.pone.0225255 (PMC6860436; doi:10.1371/journal.pone.0225255)
Supplement: Attachment — Submitted filename: responses to reviewer.docx [file pone.0225255.s003.docx]

**Response to reviewers**

**Reviewer #1**: This paper has gone though a number of revisions as the authors sort out exactly how cortisol was measured at their institution. Now they have confined their analysis to the old cortisol I assay and present an entirely new set of results.

**1. While I accept the authors' statistical analysis, I do not accept the conclusion that a cortisol done at any time of the morning is inherently superior and a more accurate predictor of adrenal insufficiency than a 0800h cortisol. It makes no sense to me how in the "normal" group, the 0800h cortisol was numerically lower than the basal cortisol (Table 1). I do agree that it is feasible to establish cortisol levels at different times of the day above and below which an ACTH stimulation test is not required to rule in or rule out adrenal insufficiency.**

We agree with the reviewer that it looks strange that the data in the normal adrenal response group showed that 0800h cortisol was lower than the basal cortisol. We re-checked the raw data and the entire data set again and found that the data shown in **Table 1,** although perhaps unexpected is correct.

Regarding the conclusion, we have modified the sentence in the abstract and have removed comparative words like “superior” and “higher”. We have further noted that basal cortisol can be used as a screening option to diagnose AI as you suggested. Other sentences related to this issue have been similarly modified (**L203, 209, 211, 245, 256, 247-248, 259**).

The title has been changed to “Diagnostic Performance of Basal cortisol Level at 0900-1300h in Adrenal Insufficiency” and the word “higher diagnostic performance” has been removed.

**2. As outlined in my previous comments, the proposed cut-offs for "morning" and "basal" cortisol lie well within the overlapping coefficients of variation of the assay.**

Thank you for mentioning this. We have modified **Table 3** and the interval of the cut-off levels have been changed to more than 10% of the prior cut-off level based on the coefficient of variation of the assay or 25 nmol/L, as appropriate. The statement in the statistical anlysis section has been modified accordingly (**L118-L119**).

**The analysis is based on an assay no longer in use and cannot necessarily be extrapolated to the new cortisol II assay.**

Thank you for your comment. We have included this issue in the Discussion section (limitations). At least, our results can be used as a guide for the future studies using the second generation cortisol assay (**L249-L257**).

**3. While acknowledged in the Discussion, mixing the low and standard dose ACTH test and using the same cortisol cut-off for each is a weakness and source of inaccuracy.**

According to the meta-analysis from Ospina NS, *JCEM 2016* (ref. #19), no significant difference in diagnostic accuracy between LDT and HDT has been demonstrated when the same cut-off point is used. However, there are still some debate about this issue.

In our study, we used ACTH dosage as one of the potentially counfounding factors and adujsted for it using multivariable regression analysis. That adjustment somewhat reduces the confounding potential related to the different ACTH dosages. We have included this potential source of diagnostic inaccuracy as a limitation in the Discussion section (**L271-272**).

**4. In the Abstract (Results), please change the cortisol units from mcg/dL to nmol/L - should read 350 nmol/L**

Thank you. The units have been changed on **L32**.

**5. In Table 1, please remove the decimal points when examining cortisol and other parameters (inappropriate to measure cortisol to 2 decimal points). Furthermore, the way the data are now presented, mean and range would be more illustrative than mean and SD.**

Thank you. The data are now shown with one decimal point and are now presented with mean and range as suggested (**Table 1**). The Statistical Analysis and Results sections have been modified accordingly (**L111 and L128**)

**Reviewer #2**:

**L.90-91
Those who had serum morning (0800 h) cortisol levels that fell into intermediate levels of 83-499 nmol/L (3-17.9 μg/dL) proceeded to either LDT or HDT.**

**As a retrospective study, and in light that the cut-offs have changed during the review process, it is misleading to state that patients were not subjected to a LDT/HDT. Rather write that these cases were classified as AI/non-AI.**

Thank you. The sentence has been modified as suggested (**L89-L90**). **L 246-247 Hence, in terms of using the proposed cut-off levels, the basal cortisol tests appear to be preferable over the 0800 h morning cortisol tests to facilitate AI diagnosis.

I understand my co-reviewers concerns on the question of morning cortisol vs. basal cortisol. There is no considerable difference between these two measurements. Also, clinical application is questionable, as slot for blood draw need to be filled anyway. Therefore, I suggest rephrasing above lines (and similar ones) into a more conciliatory tone. For instance, one could stipulate that the basal cortisol is as good as the morning cortisol, and that there is no specific need for drawing distinct morning cortisol values.**

Thank you for the suggestion. We have rephrased the conclusion in the abstract as you suggested and the title of the manuscript has been changed to “Diagnostic Performance of Basal cortisol Level at 0900-1300h in Adrenal Insufficiency”. Other sentences related to this issue have been modified (**L203, 209, 211, 245, 255, 247-248, 259**). We have used the phrase “statistically higher diagnostic performance,” but in describing the clinical implications we have adopted a more concilialtory tone, stating that basal cortisol level can be employed as a screening option to diagnose AI.

**Furthermore, this issue could be alleviated by updating figure 2 with 95% confidence bands around the ROC curves.**

Thank you for the suggestion. The 95%CI bands for ROC curves have been added in **Figure 2.**

**Table 1
As your paper is based on the reliability question of basal cortisol, it might be prudent to introduce a row for the time of blood draw in table 1**

Thank you for the suggestion. Unfortunately, as we mentioned in our response to comments on the first version of the manuscript, data regarding the specific time of the test is not available. We have included this issue in the Limitations section (**L281-L283**).
